# Supplementary figures and images for: Genome-Wide Comparative Analysis of Five Amaranthaceae Species Reveals a Large Amount of Repeat Content
Source: Plants (Basel). 2024 Mar 13;13(6):824. doi: 10.3390/plants13060824 (PMC10975975; doi:10.3390/plants13060824)

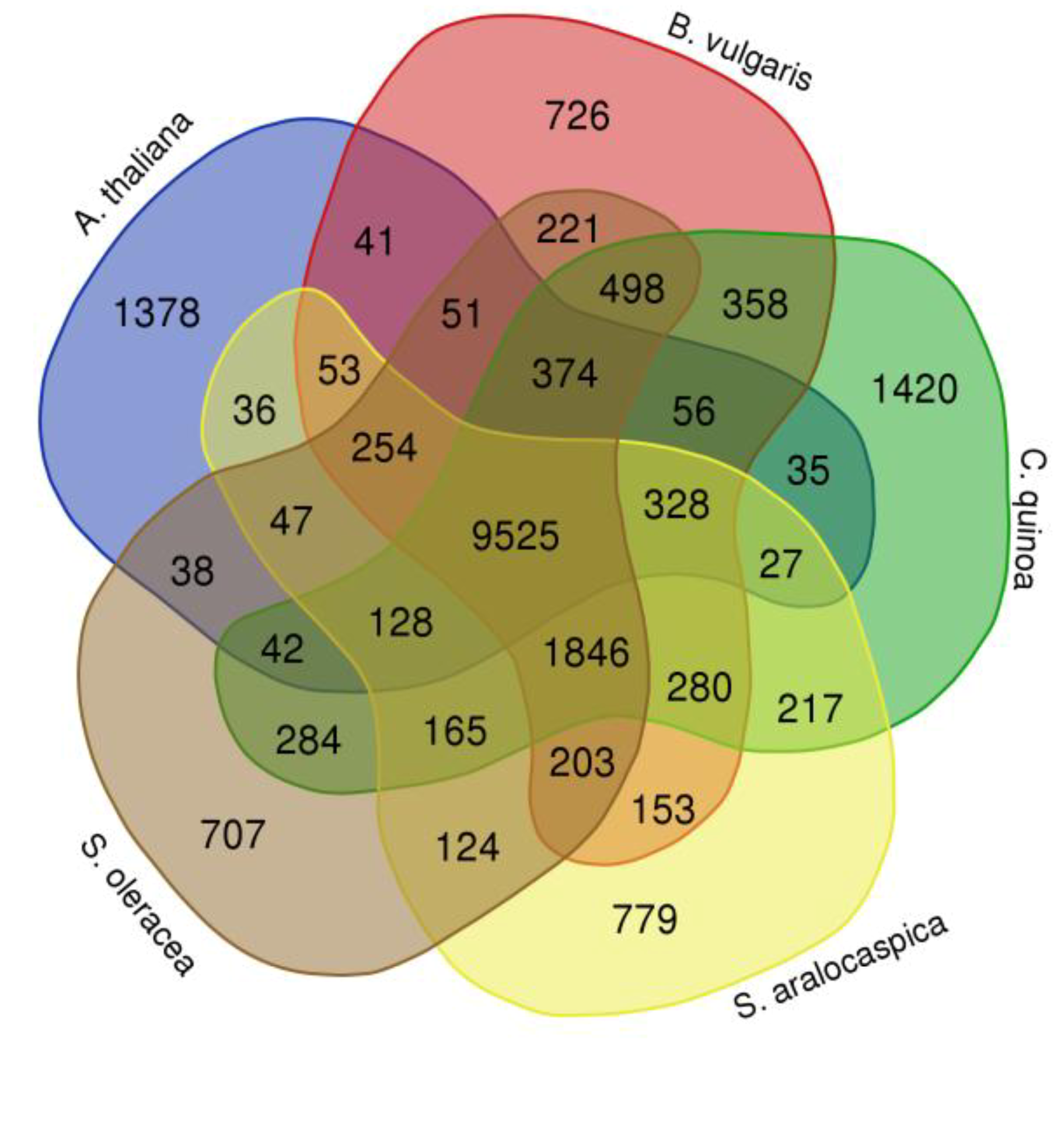

Supplement: Supplementary file 1 [file plants-13-00824-s001.zip › Figure S1.tif]

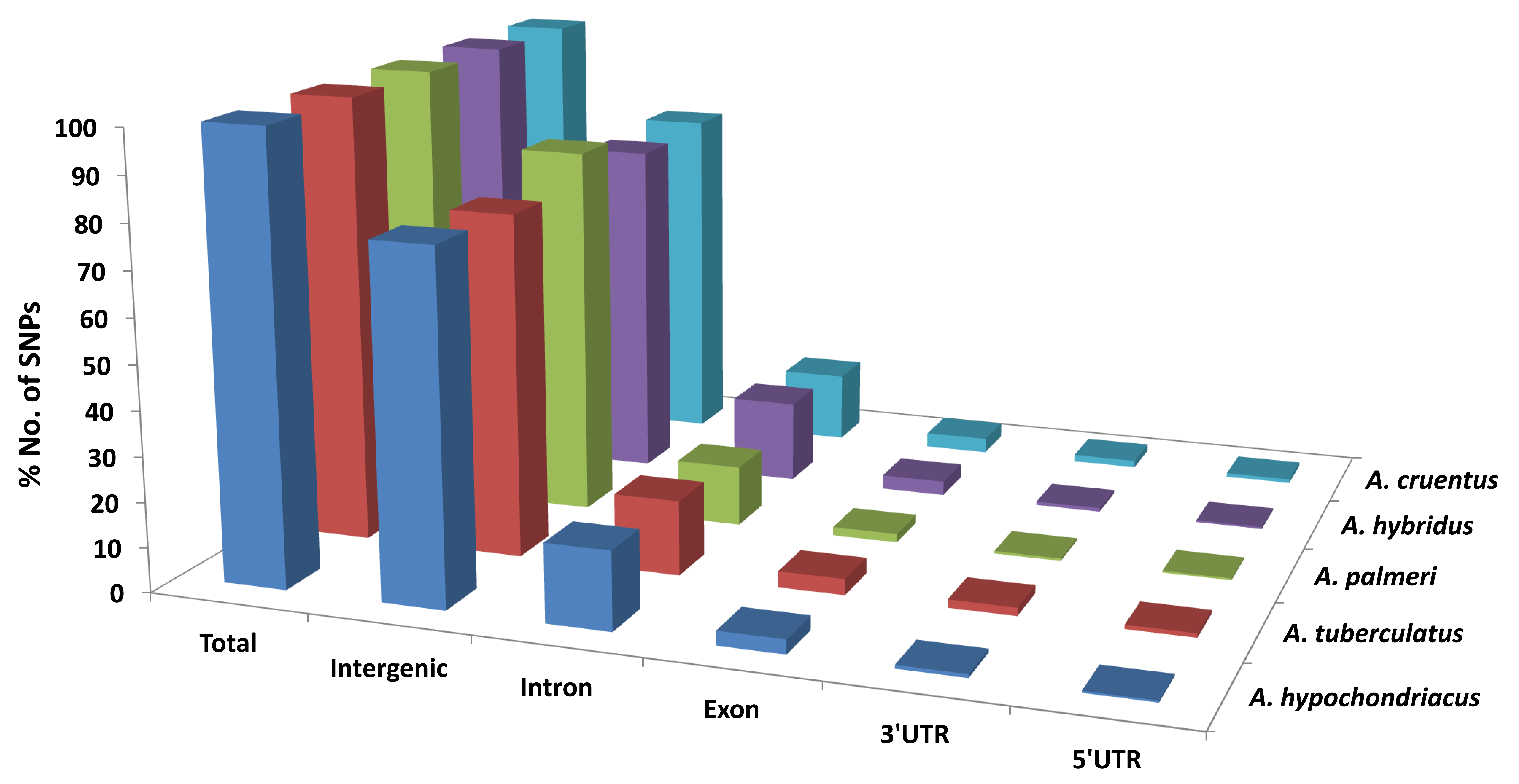

Supplement: Supplementary file 1 [file plants-13-00824-s001.zip › Figure S2.tif]

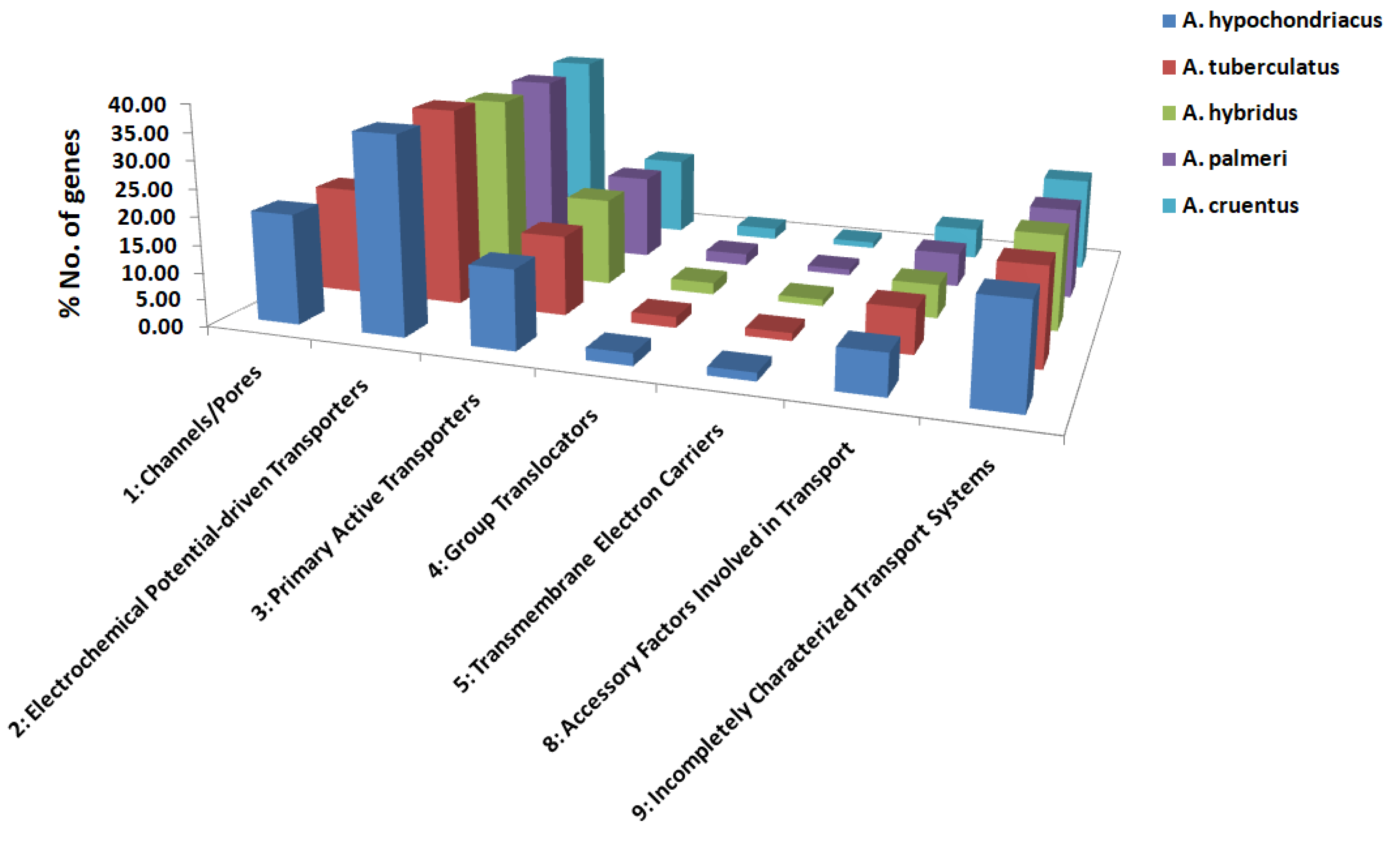

Supplement: Supplementary file 1 [file plants-13-00824-s001.zip › Figure S3.tif]

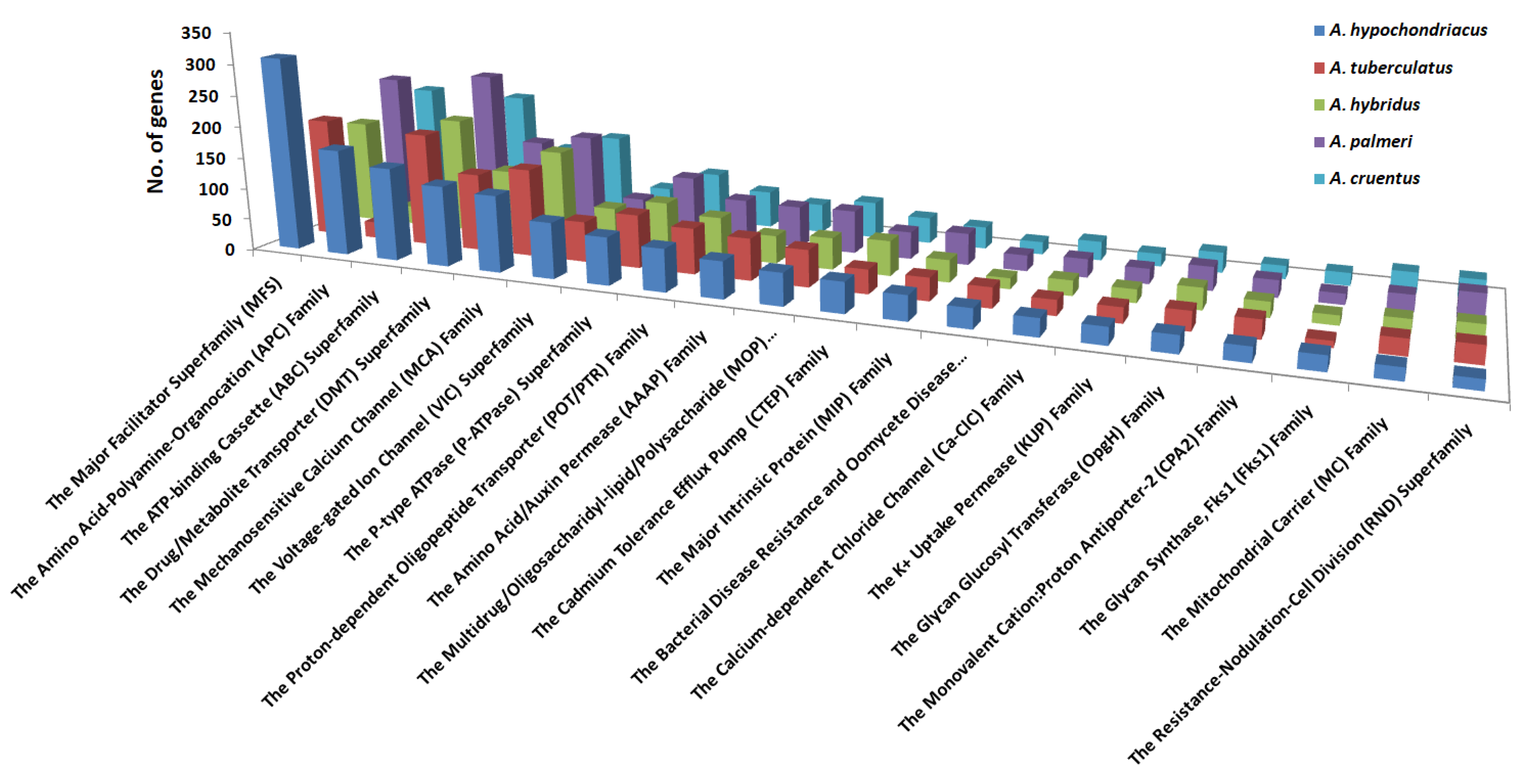

Supplement: Supplementary file 1 [file plants-13-00824-s001.zip › Figure S4.tif]
